# Effectiveness of cross‐sector collaboration in strategy implementation and impact: Evaluation of the NSW Skin Cancer Prevention Strategy 2016–2022

**DOI:** 10.1002/hpja.934

**Published:** 2024-12-12

**Authors:** Gabriela Mercado, Irina Tupanceski, Nicola Scott, Nikki Woolley, Amanda Jayakody, Kate Reakes, Sarah McGill, Tracey O'Brien

**Affiliations:** ^1^ Screening and Prevention Division The Cancer Institute NSW Sydney Australia; ^2^ Medicine and Health UNSW Sydney Australia

**Keywords:** behaviour change, collaboration, evaluation, health strategies, public health initatives, skin cancer prevention

## Abstract

**Issues Addressed:**

Australia continues to have one of the highest rates of skin cancer in the world. In NSW, melanoma is the third most common cancer diagnosed. At least 95% of skin cancers are caused by overexposure to ultraviolet radiation (UVR) from the sun, making it a highly preventable cancer. The NSW Skin Cancer Prevention Strategy (the Strategy) aims to reduce overexposure to UVR through collaborative efforts across government and non‐government partners and the private sector. An evaluation was required to inform the Skin Cancer Prevention Strategy 2023–2030 development and implementation.

**Methods:**

The Strategy was evaluated using a mixed‐methods approach, which included both process and outcome evaluation measures. Measures included population and campaign tracking surveys, administrative datasets, a document review of progress reports and meeting minutes, and semi‐structured interviews and workshops with stakeholders.

**Results:**

The evaluation outlined activities and achievements under each of the Strategy goals. Findings demonstrated improved understanding and awareness of sun protection policies and guidelines, improved access to adequate shade, increased measurability of shade availability and adequacy, and increased adoption of sun protection behaviours.

**Conclusion:**

Cross‐agency collaboration and commitment were a key strength of the Strategy. The continued prioritisation of settings (e.g. schools, workplaces, etc.) and populations outlined in the Strategy were supported by the evaluation's findings. Additional focus areas for the new Strategy were highlighted, including secondary prevention or early detection. Continued skin cancer prevention activities, including social marketing campaigns and public education programs, are essential to ensure the adoption of sun protection behaviours by priority populations.

## 1 INTRODUCTION

Australia has the highest incidence of melanoma in the world.^1^ Skin cancer, including melanoma and keratinocyte cancers, is the most common form of cancer diagnosed in Australia every year.^2^ This costs Australia's health care system $1.7 billion annually.^3^ From 2016 to 2021, melanoma was the third most common cancer in NSW, the same ranking as the previous 5‐year period.^4^ Yet, skin cancer is a highly preventable cancer. At least 95% of melanoma skin cancers and 99% of non‐melanoma skin cancers are caused by overexposure to ultraviolet radiation (UVR) from the sun.^2^

Over‐exposure to UVR can be reduced through five sun protection behaviours, summarised in health promotion campaign slogans: ‘slip on sun protective clothing, slop on SPF 30 or higher sunscreen, slap on a broad‐brimmed hat, seek shade, and slide on sunglasses’.^5^ These behaviours are most effective when they are consistently applied on days when the UV index is 3 or above, which can be year‐round in Australia.^6^ Skin cancer prevention agencies communicate to the public that over‐exposure to UVR can occur in settings such as schools, workplaces, sporting environments or during incidental exercise.

Prior to the establishment of the Cancer Institute NSW, there were state‐wide Skin Cancer Prevention Strategic Plans developed by the NSW Cancer Council and the NSW Department of Health. These plans aimed to reduce the incidence of skin cancer and associated morbidity and mortality by reducing overexposure to UVR through a coordinated and strategic approach.^7^ The NSW Skin Cancer Prevention Strategy 2012–2015 was developed following an evidence review and a rigorous consultation process with key skin cancer prevention experts in NSW, Australia, and New Zealand. This Strategy established a program of work to increase UVR protection policies, adoption of sun protection behaviours, shade provision and strategic research. A key outcome of this Strategy was a ban on commercial solaria in NSW and across Australia.^8^

The NSW Skin Cancer Prevention Strategy 2016–2022 (the Strategy) continued the focus on policy and sun protection behaviours with an increased emphasis on shade. To guide the implementation of the Strategy, government and non‐government representatives from health, education, sport, workplaces and shade‐related sectors (including professional planners and designers) worked together on an advisory committee and five settings‐focused working groups. The Strategy's goals were to: (1). increase implementation of comprehensive, effective sun protection policies and guidelines, (2) improve access to adequate shade and (3) increase adoption of sun protection behaviours. Priority populations for activities under the Strategy were children, adolescents and young people and men over 40 years of age, based on research showing the damaging impact of overexposure to sun in early life,^6^ the lower adoption of sun protection behaviours by adolescents^9^ and the increases in the incidence of melanoma in males over 40 years.^8^

Overall, melanoma incidence rates have remained unchanged over the past 10 years in NSW.^4^ An important step in working towards a reduction in the incidence of melanoma and other skin cancers is learning from strategy evaluations and using evaluation findings to inform future implementation. This brief report describes the evaluation of the 2016–2022 Strategy, covering process and outcome measures, with the overall purpose of informing the development of an NSW Skin Cancer Prevention Strategy for 2023–2030.

## 2 METHOD

### 2.1 Evaluation design

**FIGURE 1:**
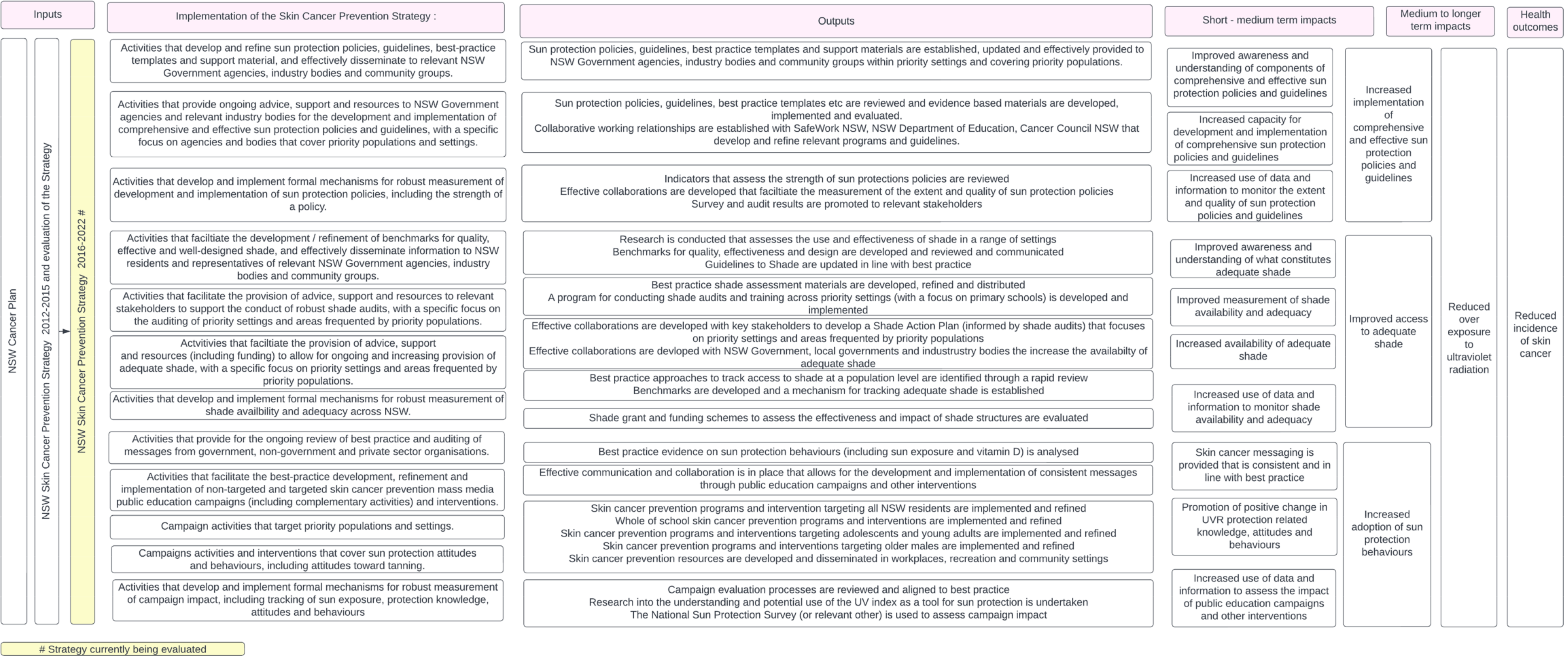
NSW Skin Cancer Prevention Strategy 2016–2022 – Logic model.

A mixed methods approach encompassing both process and outcome evaluation measures was undertaken. External consultants were engaged to conduct the evaluation. In line with best practice evaluation planning,^10^ a logic model was developed (see Figure 1), which defined the scope and parameters of the evaluation, guided the development of the evaluation questions and framework, and enabled the identification of relevant evaluation data sources.

### 2.2 Qualitative measures

A review of documents relating to the implementation of the Strategy (progress reports, a mid‐term review and working group meeting minutes) was conducted. Other documents relating to sun protection policies and guidelines were assessed, relevant shade research and evaluation activities, and an evidence scan on skin cancer in Aboriginal communities was completed. Semi‐structured interviews were conducted with key informants (*n* = 20) from organisations involved in the implementation of the Strategy, and workshops (*n* = 5) were conducted with the Skin Cancer Prevention Advisory Committee and each of the Strategy working groups (public education, schools, shade, sport & recreation and workplaces).^11^ Informants were asked questions on the achievements of the Strategy, barriers and enablers, the changing environment for skin cancer prevention, and lessons for the future.

### 2.3 Quantitative measures

Several secondary data sources provided relevant outcome indicators related to each of the Strategy goals and outcomes of the Strategy. Data was included from the biennial Sun Protection Module in the NSW Population Health Survey 2016 and 2018, and the Skin Cancer Online Tracking Survey 2016 to 2022, primarily used to monitor and evaluate Skin Cancer Prevention Campaigns implemented by the Cancer Institute NSW. Skin cancer‐related workplace claims from the State Insurance Regulatory Authority (2012–2022) were also included.

### 2.4 Data analysis

The outcomes of the analysis of the qualitative and quantitative data sources were used to evaluate progress against each of the Strategy's three goals, focusing on achievements and specific activities, initiatives, and programs. Two sample *z*‐tests were used to compare proportions, and a *p*‐value of less than 0.05 was considered statistically significant.

## 3 RESULTS

### 3.1 The governance framework supports Strategy implementation

The evaluation found the membership and resources provided by the Cancer Institute NSW and partner organisations was a significant achievement reported by all key informants. Findings from the interviews and document review found the governance structure, through the working groups and the Advisory committee, functioned as a knowledge broker, facilitated consultation with a broad range of stakeholders through the memberships, supported partnerships across organisations and enabled communication and collaboration between sectors. One informant stated, ‘The working groups have been great resources to us to assist with [our work]… It's the strategic relationship and networks that have built up personally [and] organisation to organisation’. The cross‐sector collaboration was underpinned by the governance and resulted in effective and efficient use of resources to achieve outcomes. One barrier noted by informants was the capacity of participating stakeholders to engage in the governance structure and implementation over the term of the Strategy.

### 3.2 Short‐term outcomes of the strategy's goals

Table 1 outlines key activities and achievements under each of the Strategy goals. Goal 1 (Implementation of comprehensive and effective sun protection policies and guidelines) activities led to an improved understanding and awareness of sun protection policies and guidelines across priority sectors and priority populations. Analysis of the skin cancer‐related claims showed an increase in claims from July 2012–June 2017 to July 2017–May 2022 which suggests a greater understanding of the risk of sun exposure within the workplace setting.

**TABLE 1:** A summary of key activities and achievements of the 2016–2022 Skin Cancer Prevention Strategy.

Goals	Activities	Key achievements
Goal 1: Implementation of comprehensive and effective sun protection policies and guidelines	Develop and refine sun protection policies, guidelines, best practice templates and support material and effectively disseminate to relevant NSW Government agencies, industry bodies and community groups.	Submissions were made to NSW councils regarding their Local Strategic Planning Statements (LSPSs). An evaluation of the impact found that of the 111 submissions, *n* = 66 contained at least one reference to shade. Engagement with NSW primary schools to promote sun protection policies and practices through the SunSmart Program. The 2021 SunSmart evaluation noted engagement with over 2000 schools and evidence of sun protection policies being implemented. Increased adoption of sun protection policies by State Sporting Organisations (SSOs). An assessment of the policies found that most focus on heat management and increasing the inclusion of UVR protection.
	Provide ongoing advice, support and resources to NSW Government agencies and relevant industry bodies for the development and implementation of comprehensive and effective sun protection policies and guidelines, with a specific focus on agencies and bodies that cover priority populations and settings.	
	Develop and implement formal mechanisms for robust measurement of development and implementation of sun protection policies, including the strength of a policy.	
Goal 2: Improve access to adequate shade	Develop and refine benchmarks for quality, effective and well‐designed shade, and effectively disseminate information to NSW residents and representatives of relevant NSW Government agencies, industry bodies and community groups.	Establishment of the ShadeSmart Program, which supports landscape architects and other built environment professionals in improving their awareness, understanding, and knowledge of how to design for quality shade for UV and heat protection. The 2022 ShadeSmart Awards was introduced under the AILA Landscape Architecture Awards Program. These awards support the ongoing prioritisation of sun protection amongst landscape architects and other design professionals. A partnership was formed to retrofit an existing playground in Western Sydney. This project increased usability, UV radiation protection and surface and ambient temperature. The Benchmarking Shade in NSW Playgrounds project made significant contributions to measuring shade availability and adequacy in NSW. Over 2500 playground audits were completed in 91 local government areas, providing state‐wide data about shade and sun protection in NSW. Playground users expressed a desire for greater built and natural shade.
	Provide advice, support, and resources to relevant stakeholders to support the conduct of robust shade audits, with a specific focus on auditing priority settings and areas frequented by priority populations.	
	Provide advice, support, and resources (including funding) to ensure ongoing and increasing provision of adequate shade, with a specific focus on priority settings and areas frequented by priority populations.	
	Develop and implement formal mechanisms for robust shade availability and adequacy measurement across NSW.	
Goal 3: Increased adoption of sun‐protection behaviours	Development, refinement, and implementation of best‐practice non‐targeted and targeted skin cancer prevention mass media public education campaigns (including complementary activities) and interventions.	Several campaigns, including Your Time in the Sun, Real Story testimonial videos, and Pretty Shady, were executed from 2016 to 2022 that informed target audiences of the dangers of UV radiation and skin cancer and offered information, support and role‐modelling to promote the adoption of sun protection behaviours. Several complementary programs target school‐aged children, including the SunSmart Program, Sun & UV at school syllabus resources, and the SunSafe Student Ambassadors program. ‘Improve your long game’ targeted sun protection behaviours across golf clubs in NSW and showed increased sun protection behaviours and attitudinal shifts. The program reached an estimated 3000 golfers.
	Targeted campaigns and interventions should cover priority populations and settings.	
	Campaigns and interventions should cover sun protection attitudes and behaviours, including attitudes towards tanning.	
	Develop and implement formal mechanisms for robust campaign impact measurement, including tracking sun exposure and protection knowledge, attitudes, and behaviours among target audiences.	
	Ongoing review of best practices and audit of messaging from government, non‐government, and private sector organisations.	

Activities under Goal 2 (Improve access to adequate shade) focussed on improvements in access to adequate shade in NSW and the measurability of shade availability and adequacy. The NSW Population Health Survey (NSW PHS) found declining proportions of people finding shade in sporting areas, 69.8% in 2016 and 65.0% in 2018 (*p* < 0.05), which is a statistically significant difference. Despite landscape design initiatives and enhancements to shade in playgrounds, there were no significant improvements in the proportion of people finding shade in public parks, 82.8% in 2016 and 80.3% in 2018 (*p* > 0.05). Similarly, results for outdoor swimming pools were consistent across both survey years, 75.8% in 2016 and 73.2% in 2018 (*p* > 0.05).

A key achievement of the Strategy under Goal 3 (Increased adoption of sun‐protection behaviours) was the delivery of successful social marketing and public education campaigns. All campaigns primarily targeted 18–24 year olds. The 2018/2019 *Your Time in the Sun* campaign evaluation results showed that 60% of 18–24 year olds who reported recognising the campaign had the intention to increase their level of sun protection, which was higher than those who did not recognise the campaign (42%, *p* < 0.05).^12^

## 4 DISCUSSION

With improvements in cancer detection and treatment, melanoma mortality rates are declining in both males and females.^4^ To reduce melanoma incidence and continue improvements in mortality, a sustained and coordinated approach to skin cancer prevention is critical. In addition to the key focus of primary prevention, secondary prevention was highlighted as a new area of focus for the 2023–2030 Strategy. The stakeholders involved in the evaluation reported that broadening the Strategy scope to incorporate activities that promote awareness of the importance of early detection and diagnosis of skin cancer would be beneficial. However, stakeholders highlighted barriers to including secondary prevention activities, such as the current capacity of general practices to perform regular skin checks, limited access to and availability of dermatologists, and limited evidence to support population‐wide skin checks.^13^

A key strength of the Strategy was cross‐agency collaboration and commitment. Maintaining the same governance structures via the steering committee and working groups to oversee and implement the Strategy is a crucial learning from the evaluation. Broadening the representation on the Skin Cancer Prevention Advisory Committee and related working groups to address some of the new areas of focus, such as secondary prevention, for the 2023–2030 Strategy can further enhance this.

The evaluation and other data support the continued prioritisation of children, adolescents, young adults and men over 40 in the 2023–2030 Strategy. A study using data up to 2014 found rates of invasive melanoma are significantly decreasing in males and females under 40 in NSW, while in situ rates increased.^14^ This increase is likely due to the early detection of skin cancer before it progresses.^14^ These trends indicate that the extensive exposure of younger cohorts to skin cancer prevention messaging throughout their life may be leading to a reduction in the burden of the disease.^14^

Additionally, the evaluation recommended focusing on all outdoor workers, irrespective of gender, as they are exposed to over three times more UV radiation than indoor workers, making UV radiation a significant workplace hazard.^15^, ^16^ This focus is also echoed in the Queensland Skin Cancer Prevention Strategy, where outdoor workers are prioritised.^17^ Due to the benefit of campaigns highlighted in the evaluation and in the literature, continuing to target priority populations with campaigns is essential to ensure sun protection behaviours are adopted, which can, over time, reduce the incidence of skin cancer.^18^

An evidence scan on skin cancer in Aboriginal communities found information to suggest Aboriginal people have a lower skin cancer prevalence and incidence compared to non‐Aboriginal people.^19^ However, there is an impact of skin cancer in Aboriginal communities, contributing significantly to hospitalisations and mortality among Aboriginal people. Further evidence suggests there are disparities in access to diagnosis and treatment for skin cancer.^20^, ^21^ Community consultation is required to create a shared understanding of initiatives and services for skin cancer prevention and screening in Aboriginal communities.

The evaluation highlighted the considerable work conducted with the education sector and workplaces and noted a need to continue working within these settings in the 2023–2030 Strategy. While sport and recreation was a priority setting in the Strategy, the evaluation found a number of activities had been undertaken in the sport setting, however recreation received less attention. Therefore, there is an opportunity in the 2023–2030 Strategy to focus more on recreation settings, working with the NSW Government, local government, and recreation providers such as event and tourism businesses. While health care was identified as a setting in the Strategy, a working group was not established due to competing priorities. The focus on secondary prevention in the 2023–2030 Strategy will likely include a corresponding emphasis on health care as a priority setting.

Several limitations of the evaluation were identified. Several data sources used in previous Strategy evaluations were not available due to the surveys being delayed or no longer being implemented. Of note, the NSW PHS data was used for 2016 and 2018, but the 2020 data was not included due to a shortened survey period for the sun protection module due to COVID‐19. This affected the sample size and limited any potential time‐series comparisons.

## 5 CONCLUSION

The findings from the NSW Skin Cancer Prevention Strategy evaluation highlighted critical areas of focus for the 2023–2030 Strategy. These included continued work to increase shade in public parks, sporting areas, and outdoor swimming pools, tailored interventions for the workplace setting and priority outdoor worker populations, campaign development and delivery to increase the adoption of sun protection behaviours and maintaining a coordinated, multi‐sectoral approach across government and non‐government organisations.

The 2023–2030 NSW Skin Cancer Prevention Strategy can build on the learnings and achievements of previous Strategies. While some goals and objectives will need to remain consistent with the previous Strategy due to the ongoing burden of skin cancer, additional areas of focus will need to be on priority populations and those at higher risk of developing skin cancer. The proposed expansion into secondary prevention (early detection) will require exploratory work to identify how workforce barriers can be addressed. Robust, routinely collected data on skin cancer prevention behaviours and outcomes must be a priority to monitor the progress and outcomes of the Strategy.

## FUNDING INFORMATION

The NSW Government funded the implementation and evaluation of the Skin Cancer Prevention Strategy, which is the subject matter of this study.

## CONFLICT OF INTEREST STATEMENT

The authors declare no conflicts of interest.

## ETHICS STATEMENT

Ethics approval was not required for this evaluation as it did not directly involve humans.

## Data Availability

The data that support the findings of this study are available from the corresponding author upon reasonable request.

## References

[1] World Cancer Research Fund (WCRF) International . Skin cancer statistics. London, UK: World Cancer Research Fund; 2023 Available from: https://www.wcrf.org/cancer-trends/skin-cancer-statistics/

[2] Australian Institute of Health and Welfare (AIHW) . Skin cancer in Australia. Canberra: AIHW; 2016 Available from: https://www.aihw.gov.au/reports/cancer/skin-cancer-in-australia

[3] Australian Institute of Health and Welfare (AIHW) . Disease expenditure in Australia 2018–19. Canberra: AIHW; 2021 Available from: https://www.aihw.gov.au/reports/health-welfare-expenditure/disease-expenditure-australia

[4] Cancer Institute NSW . Detailed Statistics: Cancer Incidence and Mortality. 2024 https://www.Cancer.Nsw.Gov.au/research-and-data/cancer-data-and-statistics/data-available-now/cancer-statistics-nsw/cancer-incidence-mortality-survival

[5] Walker H , Maitland C , Tabbakh T , Preston P , Wakefield M , Sinclair C , et al. A call to action on skin cancer prevention for Australia. Public Health Res Pract. 2022;32(1):e31452117. 10.17061/phrp31452117 35290993

[6] Heckman CJ , Liang K , Riley M . Awareness, understanding, use, and impact of the UV index: a systematic review of over two decades of international research. Prev Med (Baltim). 2019;123:71–83. 10.1016/j.ypmed.2019.03.004 PMC6534479 30844501

[7] Ferguson C , Vita P . A strategic framework for skin cancer prevention in NSW. NSW Public Health Bulletin. 2001;12(3):75–77. 10.1071/NB01022 12105616

[8] Cancer Institute NSW . NSW skin cancer prevention strategy 2012–15 evaluation report. 2016.

[9] Thoonen K , Woodhouse S , Minto C , Blane S , Talati Z . Patterns of sun protection behaviours among Australian adolescents and adults over a six‐year period. Curr Oncol. 2023;30(8):7178–7188. Available from: https://www.mdpi.com/1718-7729/30/8/520 37623001 10.3390/curroncol30080520 PMC10453427

[10] Centre for Epidemiology and Evidence, Population and Public Health Division . Study Design for Evaluating Population Health and Health Service Interventions: a guide. Evidence and evaluation guidance series. Sydney: NSW Ministry of Health; 2019.

[11] NSW Government . NSW skin cancer prevention strategy. Vol 2023. Sydney: Cancer Institute NSW; 2022 Available from: https://www.cancer.nsw.gov.au/prevention-and-screening/preventing-cancer/preventing-skin-cancer/nsw-skin-cancer-prevention-strategy

[12] Cancer Institute NSW . Skin Cancer Online Tracking Survey (SCOTS) 2018–19 Final Report. 2019 [Unpublished].

[13] Janda M , Olsen C , Mar V , Cust A . Early detection of skin cancer in Australia – current approaches and new opportunities. Public Health Res Pract. 2022;32(1):e3212204. 10.17061/phrp3212204 35290997

[14] Blazek K , Furestad E , Ryan D , Damian D , Fernandez‐Penas P , Tong S . The impact of skin cancer prevention efforts in New South Wales, Australia: generational trends in melanoma incidence and mortality. Cancer Epidemiol. 2022;81:102263 Available from: https://www.sciencedirect.com/science/article/pii/S1877782122001680 36174452 10.1016/j.canep.2022.102263

[15] Gies P , Wright J . Measured solar ultraviolet radiation exposures of outdoor workers in Queensland in the building and construction industry. Photochem Photobiol. 2003;78(4):342–348. 14626661 10.1562/0031-8655(2003)078<0342:msureo>2.0.co;2

[16] Kimlin MG , Parisi AV , Wong JCF . Quantification of personal solar UV exposure of outdoor workers, indoor workers and adolescents at two locations in southeast Queensland. Photodermatol Photoimmunol Photomed. 1998;14(1):7–11. Available from: https://research.usq.edu.au/item/q05wq/quantification-of-personal-solar-uv-exposure-of-outdoor-workers-indoor-workers-and-adolescents-at-two-locations-in-southeast-queensland 9582080 10.1111/j.1600-0781.1998.tb00002.x

[17] Skin Cancer Prevention Queensland . Skin cancer prevention Queensland: towards a future of reduced skin cancer burden for Queenslanders. 2023.

[18] Doran CM , Ling R , Byrnes J , Crane M , Shakeshaft AP , Searles A , et al. Benefit cost analysis of three skin cancer public education mass‐media campaigns implemented in New South Wales, Australia. PLoS One. 2016;11(1):e0147665. 26824695 10.1371/journal.pone.0147665 PMC4732951

[19] Haigh H , Mundy J . Review of Cancer among Aboriginal and Torres Strait Islander People In 2018. Available from: https://api.semanticscholar.org/CorpusID:53683025

[20] Heyes C , Tait C , Toholka R , Gebauer K . Non‐infectious skin disease in indigenous Australians. Australas J Dermatol. 2014;55(3):176–184. 10.1111/ajd.12106 25117159

[21] Slape DRM , Saunderson RB , Tatian A , Forstner DF , Estall V . Cutaneous malignancies in indigenous peoples of urban Sydney. J Med Imaging Radiat Oncol. 2018;63:63. Available from: https://api.semanticscholar.org/CorpusID:53567526–249. 10.1111/1754-9485.12832 30447047

